# Tinkering with the C-Function: A Molecular Frame for the Selection of Double Flowers in Cultivated Roses

**DOI:** 10.1371/journal.pone.0009288

**Published:** 2010-02-18

**Authors:** Annick Dubois, Olivier Raymond, Marion Maene, Sylvie Baudino, Nicolas B. Langlade, Véronique Boltz, Philippe Vergne, Mohammed Bendahmane

**Affiliations:** 1 Reproduction et Développement des Plantes, Université Lyon, Lyon, France; 2 Laboratoire de Biotechnologies Végétales Appliquées aux Plantes Aromatiques et Médicinales, Université Jean Monnet, Saint-Etienne, France; 3 Institut National de la Recherche Agronomique, Castanet Tolosan, France; University College London, United Kingdom

## Abstract

**Background:**

Roses have been cultivated for centuries and a number of varieties have been selected based on flower traits such as petal form, color, and number. Wild-type roses have five petals (simple flowers), whereas high numbers of petals (double flowers) are typical attributes of most of the cultivated roses. Here, we investigated the molecular mechanisms that could have been selected to control petal number in roses.

**Methodology/Principal Findings:**

We have analyzed the expression of several candidate genes known to be involved in floral organ identity determination in roses from similar genetic backgrounds but exhibiting contrasting petal numbers per flower. We show that the rose ortholog of *AGAMOUS* (*RhAG*) is differentially expressed in double flowers as compared to simple flowers. In situ hybridization experiments confirm the differential expression of *RhAG* and demonstrate that in the double-flower roses, the expression domain of *RhAG* is restricted toward the center of the flower. Conversely, in simple-flower roses, *RhAG* expression domain is wider. We further show that the border of *RhAG* expression domain is labile, which allows the selection of rose flowers with increased petal number. Double-flower roses were selected independently in the two major regions for domestication, China and the peri-Mediterranean areas. Comparison of *RhAG* expression in the wild-type ancestors of cultivated roses and their descendants both in the European and Chinese lineages corroborates the correlation between the degree of restriction of *RhAG* expression domain and the number of petals. Our data suggests that a restriction of *RhAG* expression domain is the basis for selection of double flowers in both the Chinese and peri-Mediterranean centers of domestication.

**Conclusions/Significance:**

We demonstrate that a shift in *RhAG* expression domain boundary occurred in rose hybrids, causing double-flower phenotype. This molecular event was selected independently during rose domestication in Europe/Middle East and in China.

## Introduction

**Figure 1:**
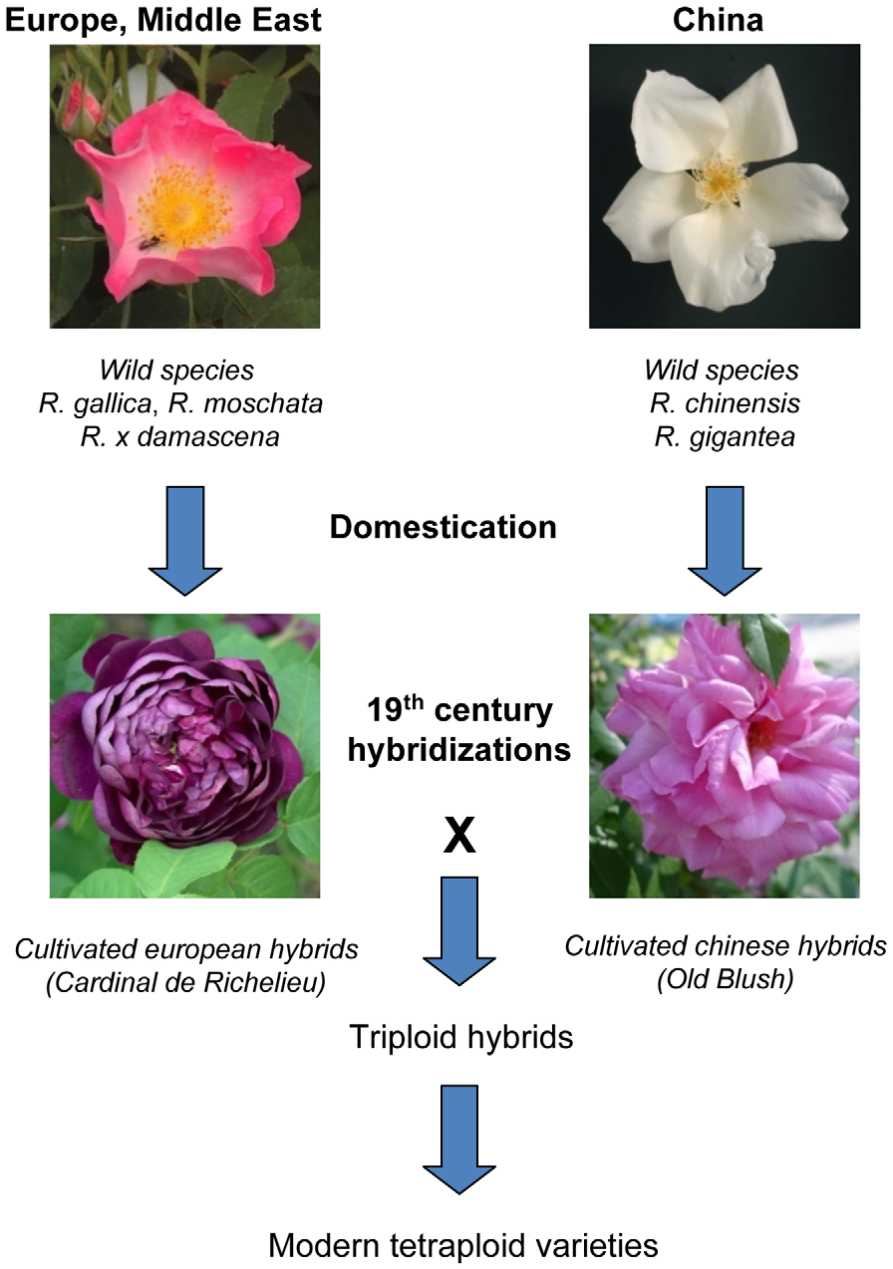
Simplified genealogy of roses. Cultivated roses originate from two main regions of domestication, *i.e.* the peri-mediterranean areas (Europe/Middle-East) and China. Double flowers were selected independently in the European and Chinese lineages. ‘Cardinal de Richelieu’ and ‘Old Blush’ represent examples of double and semi-double flower varieties in the *R. gallica* and *R. chinensis* lineages. These two gene pools were kept separated until the early nineteenth century, when they were crossed to obtain triploid hybrids and tetraploid modern varieties.

Artificial selection for certain animal and plant physical traits by breeders was first used by Charles Darwin as a surrogate to describe the natural selection process underlying evolution [1]. In recent times, the study of artificial selection processes continues to help shaping the general concepts and models for evolution [2]. In particular, the study of the genetics of crop domestication has recently made enormous progress [3]. Several essential crop characters (such as yield, plant architecture and shedding) were selected during the early phase of domestication. The current improvement phase focuses on augmented nutritional value and resistance to various abiotic and biotic stresses [3]. The molecular mechanisms controlling these processes are becoming increasingly well understood. Many of the ‘favorable’ mutations selected during domestication affect the activity or the expression patterns of master regulatory genes. Some of the best documented examples are found in grasses, where developmental genes encoding mainly transcription factors such as *TB1* and *TGA1* [4], [5], were found to be associated with domestication (see [6] for exhaustive review). In ornamental plants, flower traits such as the floral architecture, petal color and recurrent flowering are key characters that have been subjected to artificial selection pressure during the early domestication and the subsequent breeding process. Flower forms with increased number of petals (termed double flowers) were retained for their showy aspect in many domesticated plant families. In Rosaceae, for instance, spontaneous double flower forms were kept and propagated for garden ornament (*Prunus*, *Rosa*, *Potentilla…)*. Rose species were domesticated several times independently. The two major areas of rose domestication in the Antiquity were China and the peri-mediterranean area (encompassing part of Europe and Middle East), where *R. chinensis* Jacq. and *R. gallica* L. were bred and contributed predominantly to the subsequent selection process (Figure 1). In both cases semi-double (8 to 40 petals) and double flower (over 40 petals) forms were selected. There was no significant gene flow between the diploid Chinese and tetraploid European rose genotypes until the early 19^th^ century when the first triploid hybrids with reduced fertility were produced, from which our modern tetraploid hybrids arose after recurrent backcrosses (Figure 1; [7], [8]). Other species, such as *R. rugosa* Thunb., were not domesticated until the late 19^th^ or early 20^th^ centuries, and contributed to the modern breeding programs for their hardiness and disease resistance properties [7]. The 200 years of documented rose breeding history is thus a unique resource to study rose hybrids and their wild ancestors and to pinpoint molecular mechanisms that could have been selected to generate double flowers.

The genetic networks controlling floral development are extensively studied in model species such as *Arabidopsis thaliana* and are increasingly described in some non-model plants [9]. These studies led to the establishment of the ABCE model of flower development [10], [11], [12]. In this model, sepal identity is specified by A and E gene classes, petal by A, B and E gene classes, stamen by B, C and E gene classes and carpel by C and E gene classes. All of these genes (except *APETALA2*) encode MADS-box transcription factors which have been proposed to interact and form higher order protein complexes that control floral organ identity [13], [14], [15]. The C-function gene *AGAMOUS (AG)* plays a central role in specifying sexual organ identity [10], [16]. *AG* loss-of-function in *Arabidopsis* results in a shift of the boundaries of the A gene class toward the center of the flower, which transforms stamens into petals and carpels into sepals. Furthermore, flowers of the *agamous* mutant are indeterminate with new abnormal flowers reiterating in the center of the floral meristem, indicating that this gene plays a key role in floral meristem termination. The role of floral development genes is conserved in angiosperms, although increasing evidence suggests that differences in regulation, redundancy and function of these genes exist between species [9].

Here, we hypothesized that modulation of the floral key developmental genes has been the easiest way to stabilize new floral phenotypes of ornamentals during the selection process. We use two genetically cognate rose genotypes with contrasting petal numbers to investigate the possible molecular mechanisms involved in double rose flower selection. We demonstrate that the increase of petal number in roses is a consequence of a deregulation of expression of the rose ortholog of *AGAMOUS*. We provide evidence that the same event, i.e. restriction of the rose *AGAMOUS* expression domain, has been selected in double flowers during earlier events of rose domestication and at different stages of rose breeding history.

## Results

### A Mutant Approach to Study Double Flowers in Roses

**Figure 2:**
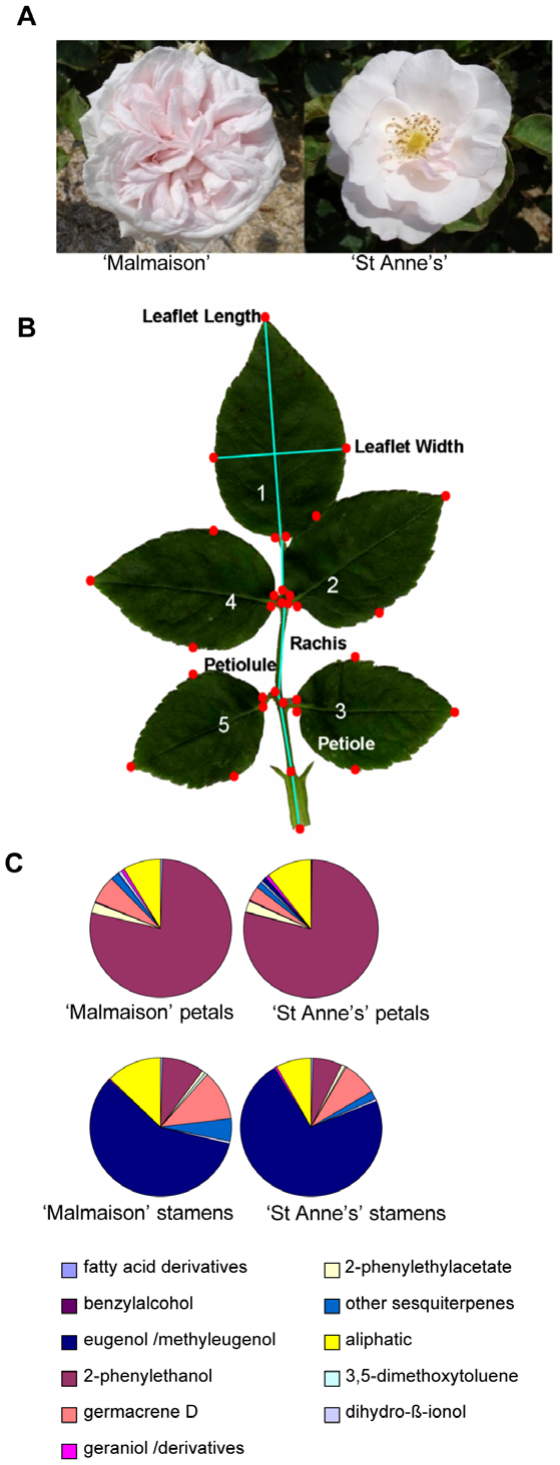
‘Malmaison’ and ‘St Anne's’ rose varieties display highly similar phenotypes, except for floral architecture. (A) Contrasting floral morphologies of *R. x hybrida* ‘Malmaison’ (double flower, left) and its genetically related variety *R. x hybrida* ‘St Anne's’ (semi-double flower, right). (B) Morphometric analysis of leaves. The photo displays a rose leaf and the 32 landmarks (red dots) that were used for measurements. No significant difference could be observed in leaf morphology between the two rose hybrids. (C) Gas Chromatography-Flame Ionization Detector (GC-FID) analysis on solid/liquid extracts of petals and stamens of *R. x hybrida* ‘St Anne's’ and ‘Malmaison’, showing that individually, the floral organs (petals and stamens) produce similar volatile compounds in both cultivars.

Sport cultivars, *i.e.* spontaneous somatic mutants, represent an interesting resource for breeders, as well as for molecular studies. It is estimated that up to 10% of cultivated rose varieties [17] are sport cultivars and they are widely used to generate new cultivars. *R*. x *hybrida* ‘Souvenir de la Malmaison’ (‘Malmaison’ hereafter) is a triploid hybrid originating from crosses between European and Chinese rose gene pools [8]. As these crosses occurred in the ‘Réunion’ (originally named ‘Bourbon’) Island, the resulting generation of triploid hybrids was designated as ‘Bourbon’ roses. ‘Malmaison’ has double flowers comprising over 100 petals (Figure 2A). *R. x hybrida* ‘Souvenir de St Anne's’ (‘St Anne's’ hereafter) is a bud-sport cultivar of ‘Malmaison’ [17]. ‘St Anne's’ cultivar has semi-double flowers with a much lower (about 10) petal number (Figure 2A). Although their floral phenotypes are conspicuously different, historical records indicate that ‘St Anne's’ derives from ‘Malmaison’ by spontaneous mutation and thus they should be nearly isogenic [17]. We confirmed that ‘St Anne's’ is a true sport of ‘Malmaison’ using four different ISSR primers that revealed identical amplification patterns between ‘Malmaison’ and ‘St Anne's’ (data not shown). Then, we examined the vegetative organ morphology and secondary metabolism activity in these two rose varieties. Both roses show similar vegetative growth patterns. Leaf morphology in ‘Malmaison’ and ‘St Anne's’ individuals was analyzed using the AAM Toolbox [18]. Thirty-two morphological measurements were obtained for each leaf (including leaflet area, length and width, petiole, rachis and petiolule length) and compared between hybrids (Figure 2B). The size and shape of all analyzed leaves from ‘Malmaison’ and ‘St Anne's’ show little variation, suggesting that both hybrids have very similar vegetative growth. We next investigated the scent composition of ‘Malmaison’ and ‘St Anne's’ since even closely related varieties can have very different fragrances [19]. Flowers of both ‘Malmaison’ and ‘St Anne's’ are heavily scented, but have slightly different scent. We conducted a headspace scent analysis and confirmed that some compounds like eugenol and methyleugenol, which give a clove scent, were present only in the headspace of ‘St Anne's’ (Table 1). We then analyzed the volatiles separately in petals and stamens by solvent extraction followed by gas chromatography. The stamens from both ‘Malmaison’ and ‘St Anne's’ produced predominantly eugenol and the petals produced mainly 2-phenylethanol, a major floral scent compound (Figure 2C). Scent signatures from both rose cultivars were identical in petals and showed only slight differences in stamens, suggesting that the mutations resulted in the ‘St Anne's’ variety only affected organ number and identity, but likely not scent. The fact that a clove scent was perceptible in ‘St Anne's’ is most probably due to the larger number of stamens in this particular variety.

**Table 1:** Major volatile compounds collected by headspace from flowers of ‘Malmaison’ and ‘St-Anne's’ and analyzed by GC-FID.

	Cultivar	
Compounds	‘Malmaison’	‘St-Anne's’
Citronellol and derivatives	1.2	1.2
Geraniol and derivatives	2.4	7.2
Delta-cadinen	0.6	1.0
β-caryophyllen	0.5	0.4
Germacrene D	11.1	13.4
Other sesquiterpenes	0.1	0.3
3,5-dimethoxytoluene	1.4	2.1
2-phenylethanol and derivatives	69.6	67.7
benzylalcohol	0.1	0.1
Fatty acid derivatives	12.9	5.4
Dihydro β-ionol	0.1	0.3
Eugenol and methyleugenol	0.0	1.0

Values represent the relative proportion of total peak area (averages of 3 different replicates).

Together, these data demonstrate that the genetic event at the origin of ‘St Anne's’ altered mainly the petal number, but did not (or in a very limited manner) impact vegetative development or floral scent.

### ‘Malmaison’ and ‘St Anne's’ Exhibit Different Floral Organ Number and Composition

**Figure 3:**
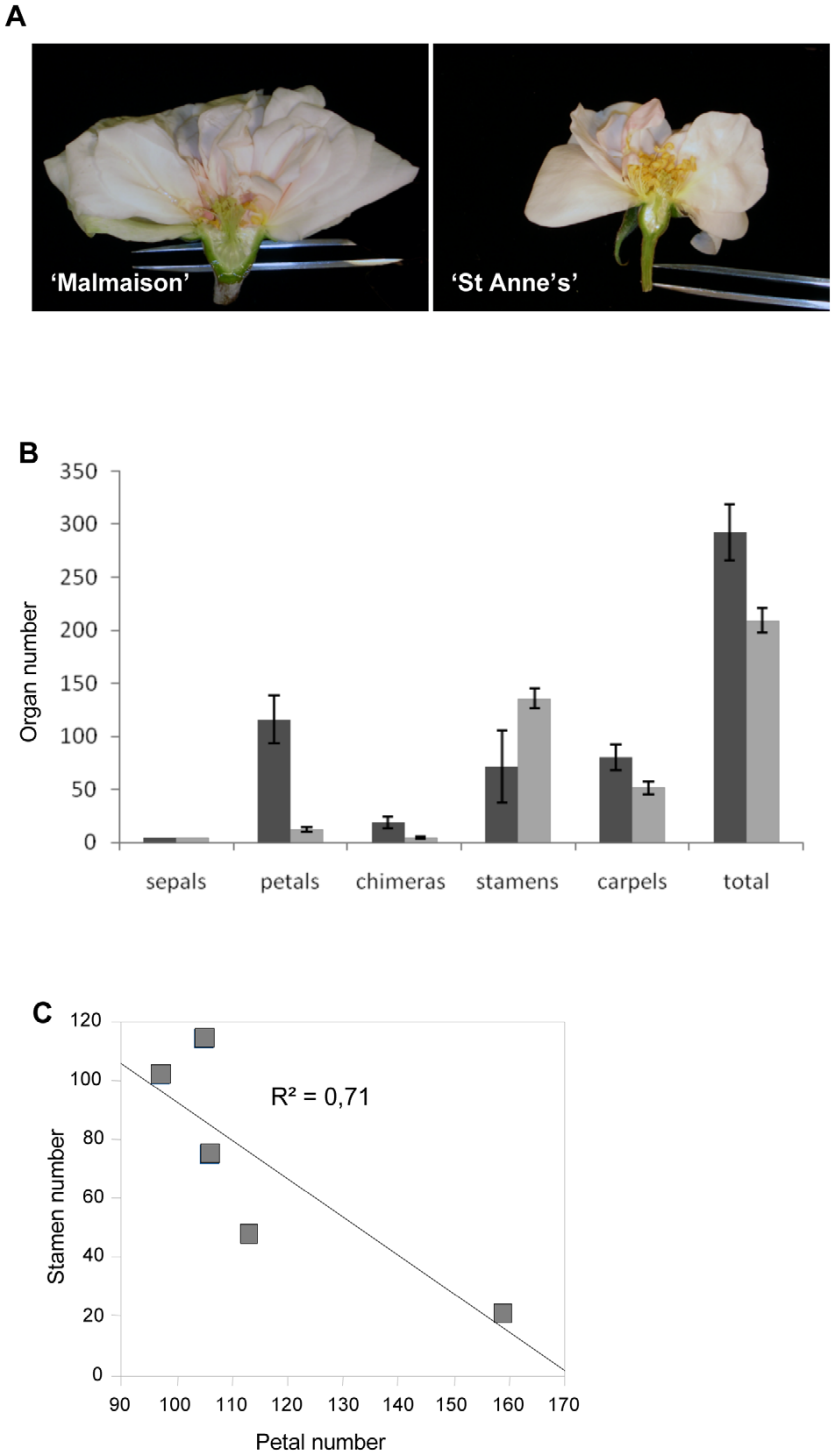
Floral organ numbers in “Malmaison” and “St Anne's”. (A) Longitudinal sections of flower in “Malmaison” (left) and in “St Anne's” (right). (B) Floral organs number in “Malmaison” (dark grey) and in “St Anne's” (light grey). Histograms represent the means obtained from 5 flowers from each hybrid. Error bars represent the standard deviation. The two rose varieties differ in two floral characters: organ identity reversions from petals in “Malmaison” to stamens in “St Anne's” and an overall decrease in total organ number. Chimeras: staminoid petals (see Figure S1). (C) Bivariate plot of petal and stamen number showing anti-correlation in “Malmaison” flowers, thus the lability of petal/stamen boundary in this genotype. Each square represents one flower. Correlation and determination coefficients are R = −0.84; R^2^ = 0.71.

Open flowers were dissected and organs were counted (Figure 3A, B, Table 2 and Figure S1). The total floral organs number was higher in ‘Malmaison’ (about 300) than in ‘St Anne's’ (about 200, Figure 3B). ‘Malmaison’ flowers had five sepals, 97 to 159 petals, 21 to 114 stamens, and 70 to 99 carpels (n = 5, Table 2). The number of stamens negatively correlates with the number of petals (R^2^ = 0.71) showing that the petal/stamen boundary is labile in ‘Malmaison’ flowers (Figure 3C). Furthermore, except for the outermost ten petals, the rest of ‘Malmaison’ petals were smaller in size compared to those of the ‘St Anne's’ (Figure S1, panels G and H), suggesting that these petals could correspond to transformed stamens. ‘St Anne's’ flowers were composed of 5 sepals, 10 to 15 petals, 123 to 148 stamens and 45 to 63 carpels (n = 5) (Figure 3B; Table 2). In contrast to ‘Malmaison’, the ‘St Anne's’ variety has much lower number of petals, but much higher number of stamens. Staminoid petals were observed in both rose genotypes, but were in a higher proportion in ‘Malmaison’ (Figure 1S panels E and F).

**Table 2:** Floral organs number in “Malmaison” and “St Anne's”.

		Malmaison	St Anne's
Sepals	Min-max	5–5	5–5
	mean	5	5
	St. dev.	0	0
Petals	Min-max	97–159	10–15
	mean	116	12.4
	St. dev.	22	1.7
Petal/stamen chimera	Min-max	14–29	3–6
	mean	18.8	4.6
	St. dev.	5.3	1.2
Stamens	Min-max	21–114	123–148
	mean	72	135.8
	St. dev.	34	9.7
Carpels	Min-max	70–99	45–63
	mean	80.4	51.4
	St. dev.	11	6

Organs were counted using five flowers per cultivar Note the high variability of stamen and petal number in ‘Malmaison’, which reflects the lability of the petals/stamens boundary in this genotype. Conversely, in ‘St Anne's’, petal and stamen numbers are much less variable.

The drastic reduction of petal number from about 110 in ‘Malmaison’ to about 11 in ‘St Anne's’ suggests an homeotic conversion in organ identity from petals into stamens (Figure S1 panels G and H). Moreover, the difference in the total number of floral organs between ‘Malmaison’ and ‘St Anne's’ also suggests a difference in floral meristem size.

### Floral Morphogenesis at Early Stages in ‘Malmaison’ and ‘St Anne's’

**Figure 4:**
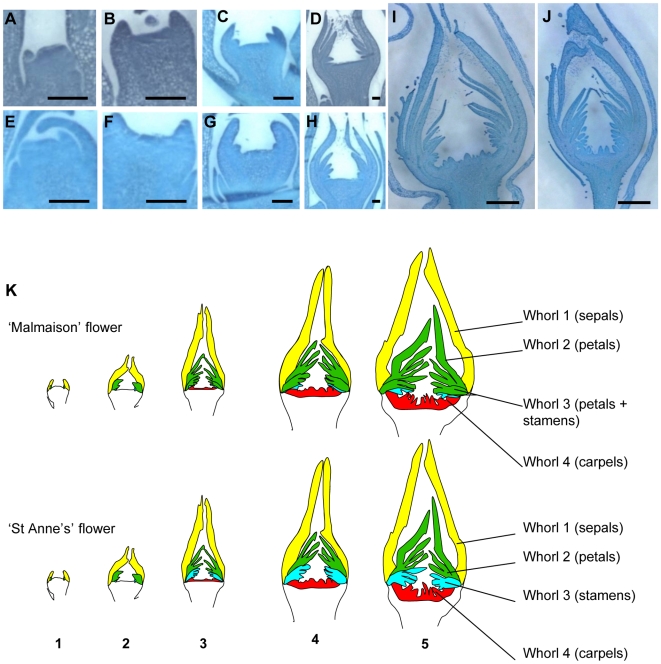
Longitudinal sections of floral meristems and flowers during floral organogenesis. (A–J) Sections (stained with toluidine blue) of “Malmaison” (A–D,I) and “St Anne's” (E–H, J) were observed, from the floral meristem stage (stage 1; A, E) until carpel formation (stage 4, I, J). Scale bar equals 150 µm for A to H and 1 mm for I and J. (K) Analysis of floral organogenesis in “Malmaison” (top) and “St Anne's” (bottom). Sepals, petals, stamens and carpels are labeled in yellow, green, blue and red colors, respectively. The different whorls composition is displayed as follows: whorl 1 comprises 5 sepals; whorl 2 is composed of the first 10 petals; whorl 3 is composed of stamens in “St Anne's” and petals plus stamens in “Malmaison”; whorl 4 is composed of carpels. Numbers 1 to 5 at the bottom define the flower development stages. Note that “Malmaison” has an enlarged floral receptacle starting from stage 4 (I).

In *A. thaliana*, floral organ identity is set up during early flower development stages [10]. To identify the stage at which flower size reduction occurs in ‘St Anne's’ as compared to ‘Malmaison’, longitudinal sections of flowers were observed at different flower development stages (Figure 4). First, we divided early flower development in roses into five distinct development stages ranging from the setting of the floral meristem to carpel primordia formation. We interpreted our observations into sketches of longitudinal sections to clearly define the early stages of floral organogenesis (Figure 4K). At stage 1, sepal primordia start to form (presumptive domain in yellow). At stage 2, the first 10 petal primordia (green) emerge. Stage 3 is different in the two genotypes: stamen primordia (in blue) start to form in ‘St Anne's’ while extra petals (in green) appear in ‘Malmaison’. At stage 4, few stamen primordia and carpel primordia (red domain) eventually form in ‘Malmaison’ while only carpel primordia form in ‘St Anne's’. At stage 5, carpels start elongating in both genotypes. To check whether there were differences in meristem size, we measured the flower diameter in sections at different stages of development. The size and shape of floral meristem were similar in ‘Malmaison’ and ‘St Anne's’ flowers at very early stages of floral development (stages 1, 2 and 3; Figure 4A–H), but diverged starting of stage 4. At stage 5, the floral receptacle in ‘St Anne's’ appeared about 20% smaller as compared to ‘Malmaison’. These data show that the difference in total floral organ number between ‘Malmaison’ and ‘St Anne's’ might be due to a difference in floral meristem size starting at floral developmental stage 4 when carpels are forming.

### Reversion of Petal Identity in ‘St Anne's’ Correlates with Differential Expression of the C-Function Gene RhAG at Early Stages of Floral Development

The early steps of flower development in angiosperms are controlled by a small set of transcription factors that trigger regulatory cascades which finally lead to floral organ identity and formation [9]. In *Arabidopsis*, loss-of-function of these genes can result into homeotic conversion of floral organs and a difference in organ number [20]. As ‘Malmaison’ and ‘St Anne's’ are nearly isogenic and exhibit petal/stamen organ identity conversions, we compared gene expression for a selection of candidate homeotic genes. We analyzed the expression of the rose B- and C-function gene homologues, as in model plants these genes were shown to be implicated in petal and stamen (B-function) and stamen and carpel (C-function) identity. We used the full length sequences that were described previously in *R. rugosa* as a primary basis for primer design [21], [22], [23]. To facilitate the reading of candidate gene names, we renamed the *MASAKO B3*, *MASAKO euB3*, *MASAKO BP* (two orthologs of the B-function genes *APETALA3*, and one ortholog of *PISTILLATA* in *Arabidopsis*), as *RhTM6* (a paleo-AP3 homolog), *RhAP3*, and *RhPI* respectively (*Rh* for *Rosa x hybrida*). Similarly, *MASAKO C* and *MASAKO D* (orthologs of the *AGAMOUS* and *SHATTERPROOF* genes in *Arabidopsis*) were renamed as *RhAG* and *RhSHP* respectively.

**Figure 5:**
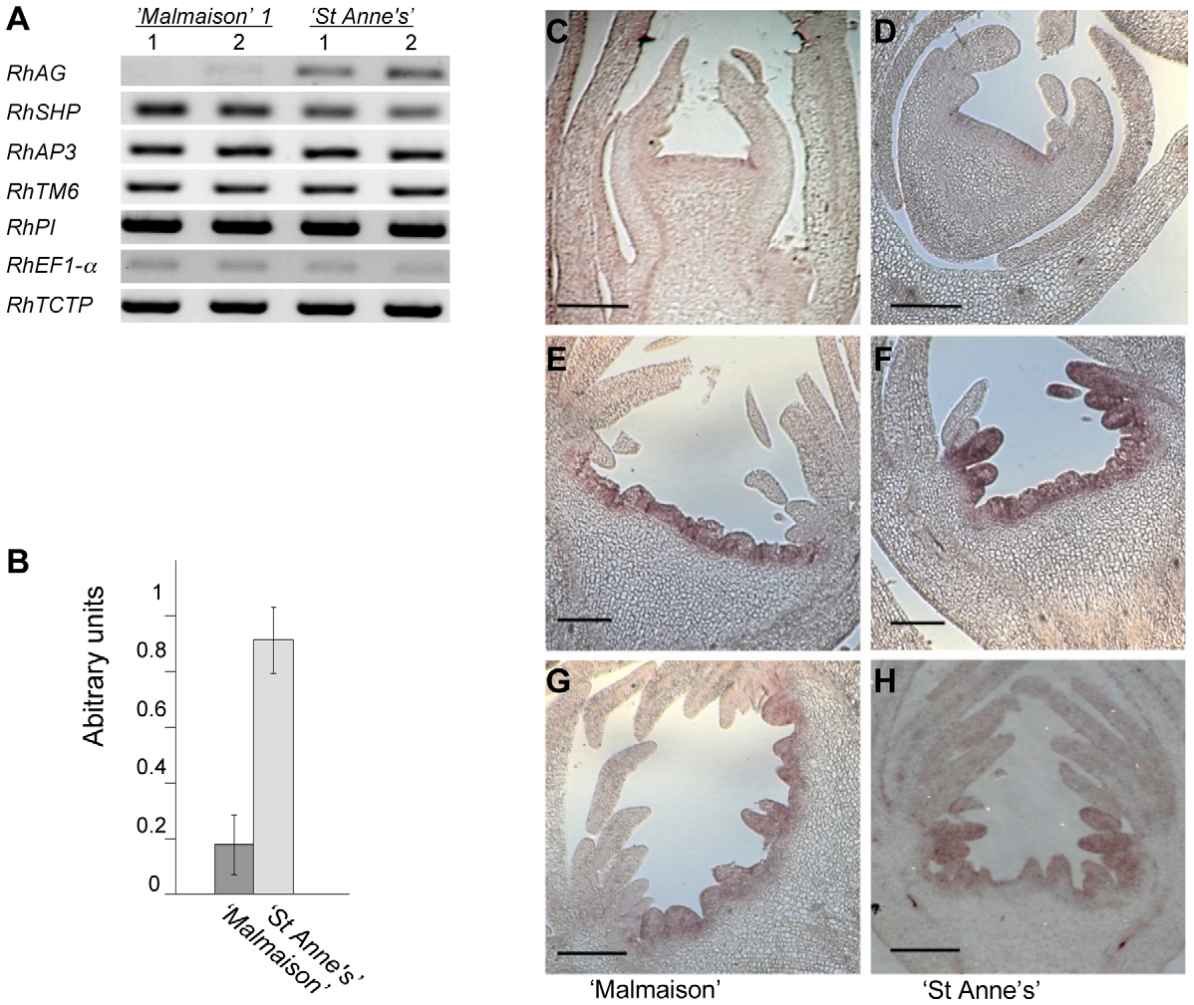
Analysis of candidate gene expression in “Malmaison” and “St Anne's” flowers at early stages of flower development. (A) RT-PCR analysis of gene expression in pools of early stages (stages 1 to 4; Figure 4 K) flowers showing differential expression of the *RhAG* gene. Two independent biological samples (1 and 2) were used. *RhEF1-alpha* and *RhTCTP* were used as housekeeping gene controls. (B) Real-time RT-PCR analysis of mRNA accumulation of *RhAG* in pools of early rose flower development stages (stages 1 to 4). *RhAG* cDNA was calibrated using both the *RhEF1-alpha* and *RhTCTP* cDNA levels. The error bars represent the standard deviation. Data from two independent biological samples were used. (C–H) *In situ* hybridizations using antisense *RhAG* sequence as probe. *In situ* hybridization on longitudinal sections of “Malmaison” flowers (C, E, G) and of “St Anne's” flowers (D, F, H) are presented. Developmental stages range from 3 to 5: (C,D) stage 3 floral buds; (E,F) stage 4 floral buds, (G,H) stage 5 floral buds. *RhAG* expression pattern is restricted towards the center of the flower in “Malmaison”. Scale bar  = 100 micrometers (C, D), 200 micrometers (E–H).

The expression of the selected genes was analyzed by RT-PCR in pools of early flowers dissected at stages 1 to 4 of flower development (Figure 5A). Among all candidate floral homeotic genes tested, only *RhAG* appeared differentially expressed between ‘Malmaison’ and ‘St Anne's’ (Figure 5A). The other tested genes, *RhAP3*, *RhTM6* and *RhPI*, displayed similar levels of expression between ‘Malmaison’ and ‘St Anne's’. Furthermore, the expression of *RhSHP*, which was proposed to act as C-function gene together with *RhAG* [21], was similar in ‘Malmaison’ and ‘St Anne's’. Real-time RT-PCR corroborated the differential expression of *RhAG* and showed that *RhAG* mRNA accumulation was 4 to 5 fold higher in ‘St Anne's’ flowers than in ‘Malmaison’ flowers (Figure 5B).

The pattern of expression of *RhAG* was analyzed using *in situ* hybridization in both hybrids. *RhAG* mRNA accumulation started to be detected in both roses at flower development stage 3 (Figure 5C, D). However, we observed a clear difference in *RhAG* pattern of expression between ‘Malmaison’ and ‘St Anne's’ at later flower development stages (Figure 5E–H). In ‘Malmaison’ flowers at stages 4 and 5, *RhAG* domain of expression remained restricted to the center of the meristem (whorl 4) and was absent from the lateral domain (whorl 3) (Figure 5E, G). In ‘Malmaison’ flowers at stage 5, the expression of *RhAG* extended slightly to the lateral domain where few primordia differentiated into stamens (Figure 5G). In ‘St Anne's’ flowers (stages 4 and 5), *RhAG* mRNA accumulation was detected in a wide cup-shaped area that extended to whorl 3 (Figures 4K, 5F and 5H). This wider area of *RhAG* mRNA accumulation was consistent with the differential expression detected by real-time RT-PCR. The lack of *RhAG* expression in the third whorl of the early developing flower in ‘Malmaison’ is associated with stamen to petal conversion. In ‘St Anne's’, *RhAG* is expressed in this domain and stamens form concomitantly instead of petals. These data show that *RhAG* expression is necessary for stamen identity and that the lack of expression of *RhAG* in the third whorl is likely responsible for double flower formation. Our data suggest that in rose flowers *RhAG* is expressed in whorls 3 and 4 and thus is involved in the determination of stamens and carpels organ identity, respectively. Therefore, the restricted expression to whorl 4 in ‘Malmaison’ likely explains the double flower formation by homeotic conversion of stamens into petals.

### Restricted Expression of the Rose *AGAMOUS* Ortholog Was Selected During Rose Domestication

To evaluate whether a similar mechanism, *i.e.* a restricted expression pattern of the rose *AG* ortholog, could have been selected to generate double roses during domestication, we compared cultivated roses to their wild-type (5 petals) ancestors. Wild-type ancestors were chosen from *R. gallica* and *R. chinensis* species, because they represent main contributors in the breeding history. We used the cultivated, recurrent blooming and simple flowered, *R. chinensis f. mutabilis* (Correv.) Rehd. (*R. chinensis* ‘mutabilis’) as model for wild-type Chinese roses. The cultivated hybrids that were studied herein have either highly double flowers with hardly any stamen (*R. gallica* ‘Cardinal de Richelieu’), or semi-double flowers (25–30 petals) with stamens (*R. chinensis* ‘Old Blush’).

**Figure 6:**
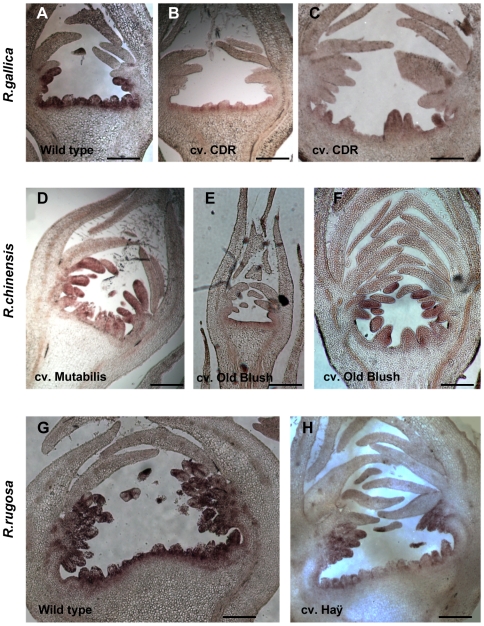
*In situ* hybridization using *RhAG* antisense probe on early stage flowers of cultivated roses and their likely wild ancestor. (A–C) Pattern of *RgAG* expression in flowers of *R. gallica* (a simple flower rose) at development stage 4 (A) and in flowers of *R. gallica* “Cardinal de Richelieu” (double flower) (B, C), at development stage 5. Note that *RgAG* expression pattern is weaker and is restricted to the central whorl (carpel domain) in “Cardinal de Richelieu”, thus in accordance to the pattern observed in “Malmaison”. (D–F) Pattern of *RcAG* expression in *R. chinensis* “mutabilis” (D) a cultivated simple flower variety that was used as a model for wild-type *R. chinensis*, and its genetically related semi-double flower rose *R. chinensis* “Old Blush” (E,F) In “Mutabilis” at stage 5 of development, *RcAG* mRNA accumulates in a wide area corresponding to whorls 3 and 4. In “Old Blush” at stage 3 of development (E) *RhAG* expression is detected in the center of the flower, whereas at stage 5 (F), *RcAG* pattern of expression is partially restricted towards the center of the flower, giving rise to semi-double corolla. (G,H) Pattern of *RrAG* expression in *R. rugosa* around stage 4 of development (G), a simple flower rose (5 petals) and (H) its genetically related hybrid descendant *R. rugosa* “Roseraie de l'Haÿ” (semi-double corolla). Scale bar (A–H)  = 200 micrometers

We analyzed the pattern of expression of the rose *AGAMOUS* orthologs by *in situ* hybridization on longitudinal sections of early developing flowers (Figure 6). In *R. gallica* ‘Cardinal de Richelieu’, *RgAG* (*Rg* for *Rosa gallica*) expression pattern was restricted to whorl 4, whereas it was present in both whorls 3 and 4 in its wild-type ancestor *R. gallica* (Figure 6A, C). These data suggest that during domestication of *R. gallica* the selection of double flower phenotype could have occurred through the restriction of *RgAG* expression.

Similarly, in flowers (stage 5) of *R. chinensis* ‘mutabilis’, we observed a pattern of expression of *RcAG* (*Rc* for *Rosa chinensis*) that closely resembled the pattern in wild-type *R. gallica* flowers, *i.e.* extended *RcAG* expression both in whorls 3 and 4 (Figure 6D). In this particular case, the wild-type pattern was probably kept during selection. However in the semi-double *R. chinensis* ‘Old Blush’, at the same stage of development, the expression of the rose *AGAMOUS* ortholog was restricted towards the center of the flower (Figure 6E, F), allowing the formation of extra petals. However, the pattern was not completely restricted to the fourth whorl (carpels) like in *R. gallica* ‘Cardinal de Richelieu’ (Figure 6C), allowing stamen formation. Next, we looked at a more recent domestication event, involving *R. rugosa* which was introduced in breeding programs in the nineteenth century. Similarly, we observed an extended *RrAG* (*Rr* for *Rosa rugosa*) expression pattern in the wild *R. rugosa* (Figure 6G), whereas this pattern was partially restricted to whorl 4 and halfway through whorl 3 in *R. rugosa* ‘Roseraie de l'Haÿ’ (a semi-double flower hybrid) allowing about 5 rows of petals to be formed (Figure 6H). Together, these data suggest that a restricted expression pattern of the rose ortholog of *AGAMOUS* has been selected independently during the domestication of *R. gallica* and *R. chinensis* to generate double flower hybrids. The same mechanism has also been selected during the introduction *R. rugosa* in breeding programs. In addition, the very double flowers had hardly any expression in whorl 3 and hardly any stamen and the semi double flowers presented an intermediate pattern. Thus, our data suggest that the severity of the double flower phenotype likely correlates with the degree of restriction of *RhAG* expression towards the center of the flower.

## Discussion

### Use of Sport Mutations in Roses

We used a mutant approach to identify candidate genes implicated in double flower formation in roses. We demonstrate that a restricted expression domain (towards the center of the flower) of the rose ortholog of *AGAMOUS* (*RhAG*) in the double flower hybrid was responsible for the transformation of stamens into petals. This restricted expression pattern of *RhAG* was selected in different species to obtain double flowers with high ornamental value during rose domestication and breeding history. Our data also demonstrate that the expression of the rose *AG* ortholog was concomitant with the establishment of stamen identity and formation in whorl 3.

Spontaneous somatic mutants have proved to be a useful resource to study various woody plant species. For example, bud sport in *Vitis* have been instrumental to identify mutations in berry color or in GA signaling [24], [25]. One can only speculate on the nature of the bud sport mutation that occurred in ‘Malmaison’ to generate ‘St Anne's’. In grape the somatic mutations that have been characterized so far are due to transposon insertions [24], [26]. In this genus, the somatic mutations often occur in one single cell which colonizes one cell layer, leading to chimeric plants [25], [27], [28]. The mutation in this study likely affects a regulatory mechanism of *RhAG* expression in whorl 3. We were unable to identify the nature of the bud sport mutation that occurred in ‘Malmaison’ to generate ‘St Anne's’. So far, cloning and sequencing of the *RhAG* cDNA and of the regulatory sequence of *RhAG* (intronic region) revealed no difference between double and simple flower roses (unpublished data). Therefore, it is tempting to speculate that either an epigenetic mutation or a mutation in an upstream regulator of *RhAG* could explain the difference between the two related varieties.

### The Rose Ortholog of *AG* Is Likely a Bona Fide C-Function Gene

In Asterids, like *Antirrhinum*, the C-function is shared by two partially redundant genes, *PLENA* (*PLENA* lineage) and *FARINELLI* (*euAGAMOUS* lineage) [29]. *PLENA* is the ortholog of the *Arabidopsis SHATTERPROOF (SHP)* gene. In *Arabidopsis, AG* performs the C-function *sensu stricto* (*i.e.* sexual organ identity and floral meristem termination), whereas *SHP* was shown to be involved later in carpel development stages [30]. Conversely, in *Antirrhinum*, *PLENA* (but not *FARINELLI*) is essential for sexual organ identity [29]. It is therefore of great interest to identify whether other Rosids share the same characteristics as *Arabidopsis* regarding the C-function. The rose ortholog of *SHATTERPROOF* (*RhSHP*) (*PLENA* lineage,[21]) was not differentially expressed between ‘Malmaison’ and ‘St Anne's’ at early stages of flower development (Figure 5), suggesting that *RhSHP* is likely not implicated in setting up organ identity at least in whorl 3 during flower development in roses. In another Rosaceae, *Taihangia rupestris*, the *SHP* ortholog is expressed in the flower slightly later than the *AGAMOUS* ortholog, suggesting that only the *AGAMOUS* ortholog might be implicated in setting up organ identity early in flower development [31]. It is thus possible that like for *SHP* in *Arabidopsis*, the rose ortholog of *SHP* may not be implicated in the initial specification of stamen identity. However, further functional analysis of this *SHP* ortholog will be necessary to draw a clear conclusion. This approach using contrasted flower phenotypes was thus useful for a better understanding of the evolution of the C-function in the genus *Rosa* and in Rosids.

The total floral organ number was also much lower in ‘St Anne's’ than in ‘Malmaison’, suggesting that the mutation may have an impact on floral meristem termination. In *A. thaliana*, *AG* is required for both floral meristem determinacy and reproductive organ identities. In *Arabidopsis* plants with reduced *AG* function (i.e. *ag* mutant or expression of antisense transgenes) the floral meristem is active for a longer period than normal, generating more floral organs than the wild-type [10], [32], [33]. Therefore, like in *Arabidopsis*, the increased total number of floral organs in ‘Malmaison’ may be associated with the observed reduced expression of *RhAG*, which in turn suggests that *RhAG* may also have a function in meristem termination in roses. However, we cannot rule out that the mutation affects other loci which control floral organ number in a *RhAG*-independent manner.

### Playing with the Sliding A/C Boundary During the Selection Process in Roses

**Figure 7:**
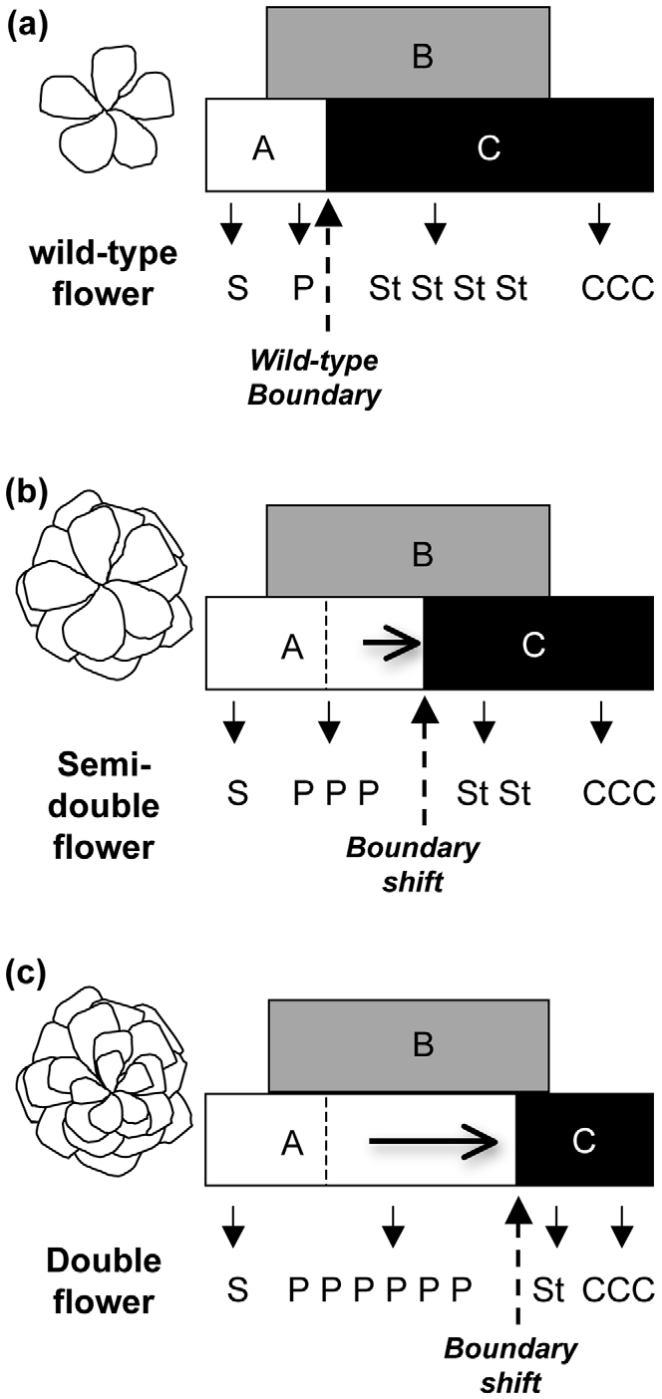
Model for selection of double roses. In wild-type roses (a), the petal/stamen boundary is very stable, as all wild species have 5 petals. In cultivated roses, the petals/stamens boundary is labile within the flowers. Breeders have tinkered with this instability of petals/stamens boundary by acting on expression domain of the rose ortholog of *AGAMOUS*, all along breeding history to select either for semi-double flowers (b) or double flowers (c).

Our data suggest that during the process of selection in the Bourbon roses (such as ‘Malmaison’ and ‘St Anne's’), breeders have empirically played with the labile petal/stamen boundary in the flower to obtain a variable number of petals. This labile boundary corresponds to the outside boundary of the rose *AGAMOUS* domain (Figure 7). Similar variation in the *AGAMOUS* pattern of expression could be observed in three species that contributed to the breeding of modern roses, *R. gallica*, *R. chinensis* and *R. rugosa*. Wild ancestor always harbored a much more extended domain of *AG* expression than their double flower descendants, where this domain was restricted towards the center of the flower. The extent of sliding for this boundary could be intermediate, generating semi-double flowers such as *R. rugosa* ‘Roseraie de l'Haÿ’ and *R. chinensis* ‘Old Blush’, or severe like in *R. gallica* ‘Cardinal de Richelieu’ (Figure 7). These results show that man could have played with this labile boundary more than once, by selecting mutations that affect the rose *AGAMOUS* pattern of expression. As Chinese and European roses have been domesticated independently, our data suggest that the same regulatory mechanism was selected at least twice independently. In the case of cereals, Paterson *et al*. (1995) suggested that in cases of convergent evolution, the same mutation could have been selected independently more than one time [34]. However, this hypothesis for cereals has been challenged during the recent years [35]. Whether the same type of mutation leading to *RhAG* misexpression was selected at many times of rose breeding history or whether different regulatory mechanisms have been selected once each time remains unclear. These mutations might be at diverse loci, but they all finally led to a “convergent developmental feature”, i.e. a restricted pattern of *RhAG* expression leading to double flower formation. Further studies on double flower formation in roses might prove valuable to understand some aspects of *RhAG* regulation, and more specifically the uncoupling of the C-function gene expression between whorls 3 and 4 of the flower. It will be necessary to search for regulators of *RhAG* and to check whether one or more are mutated in double flowers.

In summary, we investigated the molecular basis for double rose flower formation and found that a restricted expression pattern of the rose *AGAMOUS* gene correlates with the occurrence of double flowers. We demonstrate that the concept of “sliding boundary” [36] is also valid at the infra-species level and that this lability of the boundary is responsible for morphological diversity of rose flowers. Rose gardeners have tinkered [37] with this labile boundary at least twice independently during rose domestication, in Europe and in China. The molecular mechanism controlling the rose *AGAMOUS* domain of expression is currently unknown, and it will be interesting to discover whether the same regulatory mechanism or independent mechanisms were selected to generate double flowers in the two regions.

## Materials and Methods

### Plant Material

‘Malmaison’ and ‘St Anne's’ rose shrubs were purchased from Peter Beales' roses (Attleborough, Norfolk, UK); plants were planted in the field at the “Ecole Normale Supérieure-Lyon” in October 2006. *R. chinensis* cv. ‘Old Blush’ and *R. chinensis* ‘mutabilis’ were grown in the greenhouse at “Ecole Normale Supérieure-Lyon”. Independent observations and samplings were done between May 2007 and May 2009. Flowers from *R. gallica* and *R. gallica* ‘Cardinal de Richelieu’, *R. rugosa* and *R. rugosa* ‘Roseraie de l'Haÿ’ were sampled either at Lyon University or at Lyon's Botanical Garden (Parc de la Tête d'Or, Lyon) in spring 2009.

### Early Flower Morphogenesis Analysis

Inflorescences were collected from plants grown in the field at 10:00 AM. Flowers were dissected and fixed in 4% paraformaldehyde (PFA, Sigma, St. Louis, MO, USA) under vacuum infiltration and then let overnight in 4% PFA. Floral tissues were dehydrated and included in Paraplast X-tra (Thermo, Waltham MA, USA). Ten µm sections were stained using 0.5% toluidine blue and observed under a Leica MZ12 dissecting microscope.

### Morphometric Analysis

For each hybrid, 38 leaves were randomly chosen on 4 plants and scanned at 300 dpi resolution. For faster processing, images were then reduced to 60 pix/cm. Area was estimated by segmenting images with a K-means clustering analysis in a Lab color space with 2 groups using standard Matlab Image Processing Toolbox 6.2 procedures. For detailed morphometric analysis, we took advantage of the AAM Toolbox developed in Matlab [18]. A template with 32 landmarks was created in the AAM Toolbox.

### Scent Collection and Analyses

Headspace collections and solid/liquid phase extractions on petals and stamens were performed on fully opened flowers on two successive years. Each experiment was repeated at least three times on each genotype. Fragrance volatiles were extracted overnight at 4°C by immersing 1 g of tissue in 2 ml of hexane containing 40 mg.L^−1^ of camphor as an internal standard. A dynamic headspace system was also used to trap emitted volatile organic compounds according to [38]. Scent analyses were then performed according to [39]. Briefly, Gas Chromatography-Flame Ionization Detector (GC-FID) analyses were performed on an Agilent 6850 gas chromatograph equipped with a glass HP-Innowax capillary column. Volatile components were identified on the basis of retention time with authentic compounds, when available. Parallel analyses for the identification of compounds were carried out by chromatography and mass spectrometry on an Agilent 6890 gas chromatograph/mass spectrometer.

### Sample Collection for RNA Preparation

All floral meristems and flowers were dissected under a dissecting microscope. Flowers of early floral development (stage 1 to stage 4) were pooled for candidate gene expression analysis. Six to thirty flowers per stage were collected, with highest numbers for earliest (smallest in size) floral stages.

### Gene Expression Analyses

Total RNA was prepared using NucleoSpin® RNA Plant kit (Macherey-Nagel, Düren, Germany) with the following modifications. Frozen tissue was mixed with about 10% (W/V) polyvinylpyrrolidone (PVP-40, Sigma, St. Louis, MO, USA) and grinded in liquid nitrogen in Eppendorf tubes using disposable pellet pestles. RAP buffer was used for tissue lysis. All subsequent steps were performed according to the manufacturer's manual. Contaminating DNA was removed using the DNA-free™ kit (Ambion, Austin, TX, USA). Total RNA was used for cDNA synthesis using a Revert Aid M-MuLV Reverse transcriptase (Fermentas, Vilnius, Lithuania) according to the manufacturer's recommendations.

Primers (Table S1) specific to each cDNA were used for expression analysis by RT–PCR and RT-QPCR. RT-QPCR was performed with the qPCR Core Kit for SYBR Green I Quick Gold Star (Eurogentec, Liege, Belgium) using the DNA Engine Opticon® 2 Continuous Fluorescence Detection System. Reactions were run in duplicate and quantified against a relative standard curve made from a serially diluted stock cDNA containing the target sequence. Data collection and analysis were performed using the MJ OpticonMonitor analysis software (v. 3.1). Results were expressed using the relative quantification calculation method in arbitrary units as described in [40]. Relative quantification of candidate genes was performed using rose orthologs of *TRANSLATIONNALY CONTROLLED TUMOR PROTEIN* (*RhTCTP*, Genbank accession number EC587914) and *EF1-ALPHA* (*RhEF1-ALPHA*, Genbank accession number BI978089) as calibrators. These genes were identified as stably expressed in the experiment. Geometric means of the arbitrary units of the calibrator's transcripts were used to normalize the relative amount of candidate gene transcripts.

### In Situ Hybridization

*In situ* hybridizations were performed mainly as described in [41], with the following modifications: for flower fixation, vacuum was applied for 3 X 30 min. In the entire protocol, Histoclear (National diagnostics, Atlanta, GA, USA) was used instead of xylene, and H_2_O was used in place of 0.85% NaCl. Ten micrometers sections were hybridized. Hybridization was performed at 45°C overnight with a 0.1 to 0.2 ng. µL^–1^ RNA probe concentration. The first and second washes were performed at high stringencies (1× and 0.5×, respectively). The *RhAG* probe was PCR-amplified (see primer sequences in Table S1) from a *RhAG* cDNA clone from ‘Malmaison’.

## Supporting Information

Table S1 Primers used in this study.
(0.05 MB DOC)

Figure S1 Floral dissections of “Malmaison” (A, C, E, G) and “St Anne's” (B, D, F, H). (A, B): Longitudinal sections of the flowers showing that “Malmaison” has a more open floral receptacle because of the large petal number. (C, D): Stamens of “Malmaison” and “St Anne's,” respectively. Note the smaller size of the filaments in “Malmaison.” (E, F): Staminoid petals. (G, H): Petal, stamen, and carpel composition and morphology in dissected flowers, from the outside to the inside of the flower. Slashes represent discontinuities in the dissection. Note the much smaller size of the inside petals in “Malmaison.”
(4.76 MB TIF)

## References

[1] Darwin C (1859). On the Origin of Species by Means of Natural Selection, or the Preservation of Favoured Races in the Struggle for Life..

[2] Gregory T (2009). Artificial Selection and Domestication: Modern Lessons from Darwin's Enduring Analogy.. Evolution: Education and Outreach.

[3] Burke JM, Burger JC, Chapman MA (2007). Crop evolution: from genetics to genomics.. Current Opinion in Genetics and Development.

[4] Wang RL, Stec A, Hey J, Lukens L, Doebley J (1999). The limits of selection during maize domestication.. Nature.

[5] Wang H, Nussbaum-Wagler T, Li B, Zhao Q, Vigouroux Y (2005). The origin of the naked grains of maize.. Nature.

[6] Glemin S, Bataillon T (2009). A comparative view of the evolution of grasses under domestication.. New phytologist.

[7] Krussmann G (1981). The Complete Book of Roses..

[8] Martin M, Piola F, Chessel D, Jay M, Heizmann P (2001). The domestication process of the Modern Rose: genetic structure and allelic composition of the rose complex.. Theoretical and Applied Genetics.

[9] Ferrario S, Immink RG, Angenent GC (2004). Conservation and diversity in flower land.. Curr Opin Plant Biol.

[10] Bowman JL, Smyth DR, Meyerowitz EM (1989). Genes directing flower development in Arabidopsis.. Plant Cell.

[11] Parcy F, Nilsson O, Busch MA, Lee I, Weigel D (1998). A genetic framework for floral patterning.. Nature.

[12] Roeder AHK, Yanofsky MF (2001). Unraveling the mystery of double flowers.. Developmental Cell.

[13] Theissen G, Saedler H (2001). Plant biology. Floral quartets.. Nature.

[14] Saedler H, Becker A, Winter KU, Kirchner C, Theissen G (2001). MADS-box genes are involved in floral development and evolution.. Acta Biochimica Polonica.

[15] Melzer R, Verelst W, Theissen G (2009). The class E floral homeotic protein SEPALLATA3 is sufficient to loop DNA in floral quartet-like complexes in vitro.. Nucleic Acids Research.

[16] Lohmann JU, Weigel D (2002). Building beauty: the genetic control of floral patterning.. Developmental Cell.

[17] Cairns T, Young M, Adams J, Edberg B (2000). Modern Roses XI. The World Encyclopedia of Roses..

[18] Langlade NB, Feng X, Dransfield T, Copsey L, Hanna AI (2005). Evolution through genetically controlled allometry space.. Proc Natl Acad Sci U S A.

[19] Cherri-Martin M, Jullien F, Heizmann P, Baudino S (2007). Fragrance heritability in hybrid tea roses.. Scientia Horticulturae.

[20] Krizek BA, Fletcher JC (2005). Molecular mechanisms of flower development: An armchair guide.. Nature Reviews Genetics.

[21] Kitahara K, Hibino Y, Aida R, Matsumoto S (2004). Ectopic expression of the rose AGAMOUS-like MADS-box genes ‘MASAKO C1 and D1’ causes similar homeotic transformation of sepal and petal in Arabidopsis and sepal in Torenia.. Plant Science.

[22] Kitahara K, Matsumoto S (2000). Rose MADS-box genes ‘MASAKO C1 and D1’ homologous to class C floral identity genes.. Plant Science.

[23] Hibino Y, Kitahara K, Hirai S, Matsumoto S (2006). Structural and functional analysis of rose class B MADS-box genes ‘MASAKO BP, euB3, and B3: Paleo-type AP3 homologue ‘MASAKO B3’ association with petal development.. Plant Science.

[24] Walker AR, Lee E, Robinson SP (2006). Two new grape cultivars, bud sports of Cabernet Sauvignon bearing pale-coloured berries, are the result of deletion of two regulatory genes of the berry colour locus.. Plant Molecular Biology.

[25] Boss PK, Thomas MR (2002). Association of dwarfism and floral induction with a grape ‘green revolution’ mutation.. Nature.

[26] Lijavetzky D, Ruiz-Garcia L, Cabezas JA, De Andres MT, Bravo G (2006). Molecular genetics of berry colour variation in table grape.. Molecular Genetics And Genomics.

[27] Franks T, Botta R, Thomas MR (2002). Chimerism in grapevines: implications for cultivar identity, ancestry and genetic improvement.. Theoretical and Applied Genetics.

[28] Fernandez L, Doligez A, Lopez G, Thomas MR, Bouquet A (2006). Somatic chimerism, genetic inheritance, and mapping of the fleshless berry (flb) mutation in grapevine (Vitis vinifera L.).. Genome.

[29] Davies B, Motte P, Keck E, Saedler H, Sommer H (1999). PLENA and FARINELLI: redundancy and regulatory interactions between two Antirrhinum MADS-box factors controlling flower development.. Embo Journal.

[30] Causier B, Castillo R, Zhou J, Ingram R, Xue Y (2005). Evolution in action: following function in duplicated floral homeotic genes.. Curr Biol.

[31] Lu SH, Du XQ, Lu WL, Chong K, Meng Z (2007). Two AGAMOUS-like MADS-box genes from Taihangia rupestris (Rosaceae) reveal independent trajectories in the evolution of class C and class D floral homeotic functions.. Evolution and Development.

[32] Bowman JL, Meyerowitz EM (1991). Genetic control of pattern formation during flower development in Arabidopsis.. Molecular Biology of Plant Development.

[33] Mizukami Y, Ma H (1995). Separation of AG function in floral meristem determinacy from that in reproductive organ identity by expressing antisense AG RNA.. Plant Molecular Biology.

[34] Paterson AH, Lin YR, Li Z, Schertz KF, Doebley JF (1995). Convergent Domestication of Cereal Crops by Independent Mutations at Corresponding Genetic Loci.. Science.

[35] Li W, Gill BS (2006). Multiple genetic pathways for seed shattering in the grasses.. Funct Integr Genomics.

[36] Soltis DE, Ma H, Frohlich MW, Soltis PS, Albert VA (2007). The floral genome: an evolutionary history of gene duplication and shifting patterns of gene expression.. Trends In Plant Science.

[37] Jacob F (1977). Evolution and tinkering.. Science.

[38] Grison-Pige L, Bessiere JM, Hossaert-McKey M (2002). Specific attraction of fig-pollinating wasps: role of volatile compounds released by tropical figs.. J Chem Ecol.

[39] Bergougnoux V, Caissard JC, Jullien F, Magnard JL, Scalliet G (2007). Both the adaxial and abaxial epidermal layers of the rose petal emit volatile scent compounds.. Planta.

[40] Vandesompele J, De Preter K, Pattyn F, Poppe B, Van Roy N (2002). Accurate normalization of real-time quantitative RT-PCR data by geometric averaging of multiple internal control genes.. http://www.ncbi.nlm.nih.gov/pmc/articles/PMC126239/.

[41] Ma N, Xue J, Li Y, Liu X, Dai F (2008). Rh-PIP2;1, a rose aquaporin gene, is involved in ethylene-regulated petal expansion.. Plant Physiol.

