# Supplementary material for: Tinkering with the C-Function: A Molecular Frame for the Selection of Double Flowers in Cultivated Roses
Source: PLoS One. 2010 Feb 18;5(2):e9288. doi: 10.1371/journal.pone.0009288 (PMC2823793; doi:10.1371/journal.pone.0009288)
Supplement: Table S1 — Primers used in this study. (0.05 MB DOC) [file pone.0009288.s001.doc]

**Table S1 : Primers used in this study**

| Primers | *Gene* | Primer Sequence | Gene bank accession | Experiment |
| --- | --- | --- | --- | --- |
| RhAG-as1 | *RhAG* | TCAAACTAATTGAAGGGAAATCTGG | AB025644 | semi-quant. RT-PCR |
| RhAG-s1 | *RhAG* | AAAAGGGAACTTGACTTGCACAAC | AB025644 | semi-quant. RT-PCR |
| RhSHP-as1 | *RhSHP* | TTGGAGGCTTCTTGCTGATAAAAC | AB025643 | semi-quant. RT-PCR |
| RhSHP-s1 | *RhSHP* | CTGAGAGCTCTTCCCAAAAGAAATT | AB025643 | semi-quant. RT-PCR |
| RheuAP3-as1 | *RheuAP3* | GGCTGATCTGCCTCCTAAGACTCTT | AB099875 | semi-quant. RT-PCR |
| RheuAP3-s1 | *RheuAP3* | ATGGCGAGAGGTAAGATCCAGATC | AB099875 | semi-quant. RT-PCR |
| RhTM6-as1 | *RhTM6* | CGTATGATTTGCACGGATTGACT | AB055966 | semi-quant. RT-PCR |
| RhTM6-s1 | *RhTM6* | ATGGGTCGTGGGAAGATTGAG | AB055966 | semi-quant. RT-PCR |
| RhPI-as1 | *RhPI* | GCTTATTCTCGTCCTCCAGAGCT | AB038462 | semi-quant. RT-PCR |
| RhPI-s1 | *RhPI* | ATGGGGAGGGGTAAGATTGAGA | AB038462 | semi-quant. RT-PCR |
| RhEF1-alpha-as1 | *RhEF1-alpha* | AGTACCTTGTGGTCTCAAACTTCCA | BI978089 | semi-quant. RT-PCR |
| RhEF1-alpha-s1 | *RhEF1-alpha* | CACTCTTCTACTCCTTTGCCTCTCC | BI978089 | semi-quant. RT-PCR |
| RhTCTP-as1 | *RhTCTP* | TCTTAGCACTTGACCTCCTTCA | BI978618 | semi-quant. RT-PCR |
| RhTCTP-s1 | *RhTCTP* | TTTACCAGGACCTCCTCACC | BI978618 | semi-quant. RT-PCR |
| RhAG HIS rev T7 | *RhAG* | TGTAATACGACTCACTATAGGGCGTAATGCCAGTACTAACTGTGGGAGAGGTT | AB025644 | *in situ* hybridization |
| RhAG HIS for T7 | *RhAG* | TGTAATACGACTCACTATAGGGCAGGGAACTTGACTTGCACAACAATAACC | AB025644 | *in situ* hybridization |
| RhAG HIS rev | *RhAG* | GTAATGCCAGTACTAACTGTGGGAGAGGTT | AB025644 | *in situ* hybridization |
| RhAG HIS for | *RhAG* | AGGGAACTTGACTTGCACAACAATAACC | AB025644 | *in situ* hybridization |
| RhAGQ1 rev | *RhAG* | CAGCAGAAACCAGGCCAAGCATACAT | AB025644 | quant. RT-PCR |
| RhAGQ1 for | *RhAG* | CAGCCGACTCAGCCATTTCATGAGG | AB025644 | quant. RT-PCR |
| RhTCTP-R2 | *RhTCTP* | CTTGGTTGCTCCCTCAATGT | BI978618 | quant. RT-PCR |
| RhTCTP-F2 | *RhTCTP* | GATGCTGATGAGGGTGTTGA | BI978618 | quant. RT-PCR |
| RhEF1-QS1 | *RhEF1-alpha* | GGGTAAGGAGAAGGTTCACATC | BI978089 | quant. RT-PCR |
| RhEF1-QAS1 | *RhEF1-alpha* | CAGCCTCCTTCTCAAACCTCT | BI978089 | quant. RT-PCR |

semi-quant. RT-PCR: Semi-quantitative RT-PCR ; quant. RT-PCR: quantitative real-time RT-PCR
