# Supplementary figures and images for: Tinkering with the C-Function: A Molecular Frame for the Selection of Double Flowers in Cultivated Roses
Source: PLoS One. 2010 Feb 18;5(2):e9288. doi: 10.1371/journal.pone.0009288 (PMC2823793; doi:10.1371/journal.pone.0009288)

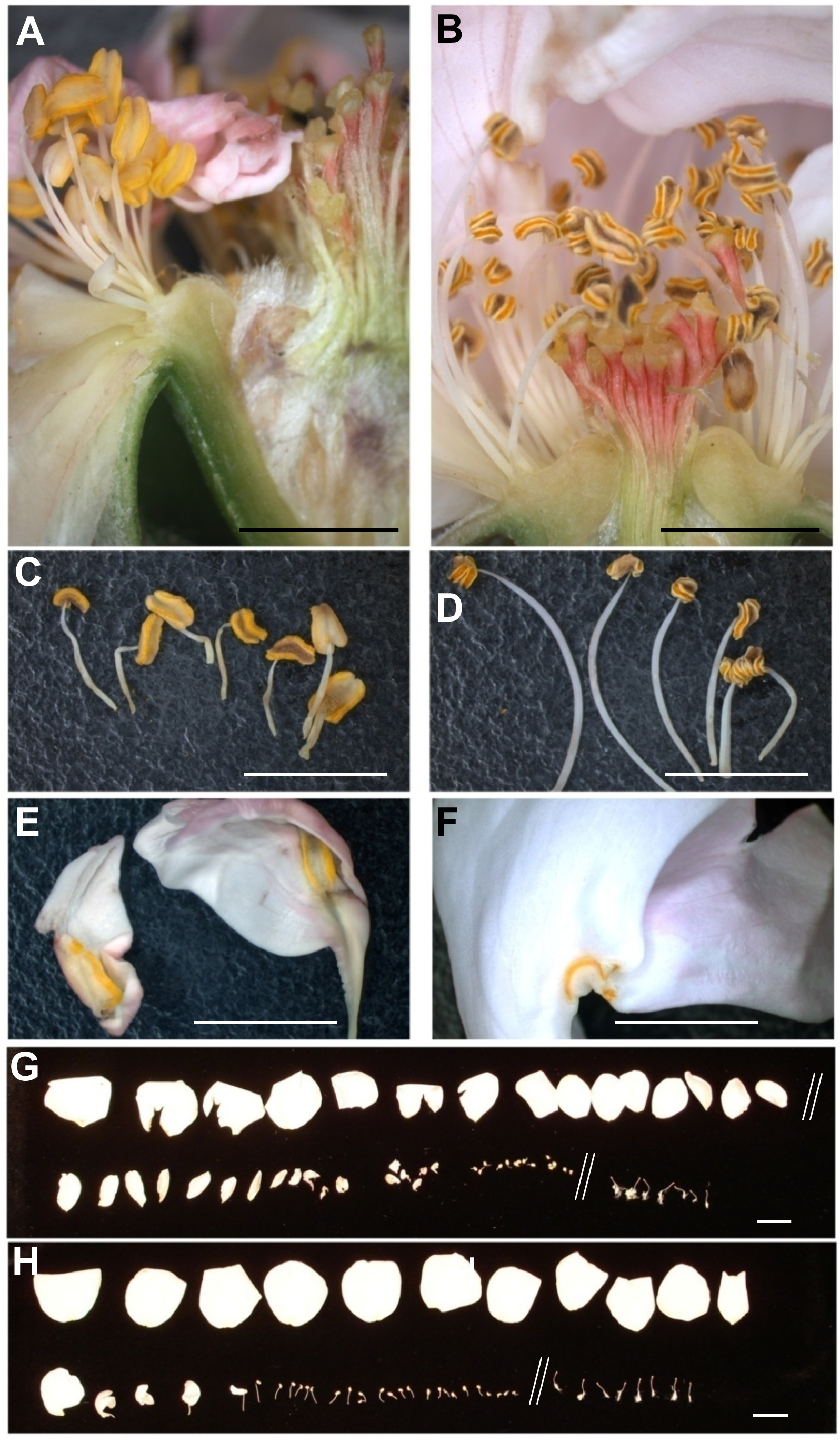

Supplement: Figure S1 — Floral dissections of “Malmaison” (A, C, E, G) and “St Anne's” (B, D, F, H). (A, B): Longitudinal sections of the flowers showing that “Malmaison” has a more open floral receptacle because of the large petal number. (C, D): Stamens of “Malmaison” and “St Anne's,” respectively. Note the smaller size of the filaments in “Malmaison.” (E, F): Staminoid petals. (G, H): Petal, stamen, and carpel composition and morphology in dissected flowers, from the outside to the inside of the flower. Slashes represent discontinuities in the dissection. Note the much smaller size of the inside petals in “Malmaison.” (4.76 MB TIF) [file pone.0009288.s002.tif]
